# Production of Phloroglucinol, a Platform Chemical, in Arabidopsis using a Bacterial Gene

**DOI:** 10.1038/srep38483

**Published:** 2016-12-07

**Authors:** Salah E. Abdel-Ghany, Irene Day, Adam L. Heuberger, Corey D. Broeckling, Anireddy S.N. Reddy

**Affiliations:** 1 Department of Biology, Program in Molecular Plant Biology, Program in Cell and Molecular Biology, Colorado State University, Fort Collins, CO 80523, USA; 2 Department of Botany, Faculty of Science, Zagazig University, Zagazig, 44519, Egypt; 3 Proteomics and Metabolomics Facility, Colorado State University, Fort Collins, CO 80523, USA

## Abstract

Phloroglucinol (1,3,5-trihydroxybenzene; PG) and its derivatives are phenolic compounds that are used for various industrial applications. Current methods to synthesize PG are not sustainable due to the requirement for carbon-based precursors and co-production of toxic byproducts. Here, we describe a more sustainable production of PG using plants expressing a native bacterial or a codon-optimized synthetic PhlD targeted to either the cytosol or chloroplasts. Transgenic lines were analyzed for the production of PG using gas and liquid chromatography coupled to mass spectroscopy. Phloroglucinol was produced in all transgenic lines and the line with the highest *PhlD* transcript level showed the most accumulation of PG. Over 80% of the produced PG was glycosylated to phlorin. Arabidopsis leaves have the machinery to glycosylate PG to form phlorin, which can be hydrolyzed enzymatically to produce PG. Furthermore, the metabolic profile of plants with PhlD in either the cytosol or chloroplasts was altered. Our results provide evidence that plants can be engineered to produce PG using a bacterial gene. Phytoproduction of PG using a bacterial gene paves the way for further genetic manipulations to enhance the level of PG with implications for the commercial production of this important platform chemical in plants.

Molecular farming is now used to produce valuable products in plants that are not only important for human and veterinary medicine but also for many industrial applications^1^,^2^,^3^. Plants, as autotrophs, have the ability to use light energy, carbon dioxide, water and simple inorganic nutrients to produce significant biomass and synthesize a diverse array of complex organic compounds that could serve as precursors for many novel chemicals^4^,^5^,^6^. Therefore, plants are cost effective and environmentally safe alternative to the chemical-based synthesis of many platform chemicals^4^,^5^. By introducing foreign genes into nuclear or chloroplast genomes high levels of non-native proteins including vaccines have been produced in subcellular compartments of plants^2^,^3^,^7^,^8^.

Phloroglucinol (PG) is the simplest member of the phloroglucinols family of organic compounds consisting of more than 700 naturally occurring derivatives that exhibit a wide array of useful biological activities^9^. The structural complexity of these compounds ranges from the parent molecule 1,3,5-trihydroxybenzene (PG) to highly complex phlorotannins with 31 PG molecules^10^. Phloroglucinol glycosides represent the most common PG derivatives and more than 50 PG glycosides have been reported from natural sources^11^. Phloroglucinol β-D-glucoside, called phlorin, is the simplest PG glucoside, which has been isolated from *Cannabis sativa, Cornus capitata* and the peel of many citrus fruits (reviewed in ref. 12).

Phloroglucinol and its derivatives have been used in pharmaceuticals, the dyeing industry, plant tissue culture and as a precursor for manufacturing of an energetic material called 1,3,5-triamino-2,4,6 trinitrobenzene (TATB)^4^,^11^,^13^. In the fruit juice industry phlorin is widely used as an orange peel marker for juice quality^12^. In addition, it has antimicrobial activity against oral bacteria^14^. The PG-chitosan conjugate inhibits the tyrosinase activity that is responsible for the browning of food, hence has a potential use in the food industry as a food preservative^15^. In medicine, PGs have various useful biological properties such as anti-inflammatory, anticancer, anti-microbial, antioxidant, and neuro-regenerative activities^9^,^16^,^17^,^18^,^19^,^20^. PG derivatives such as aspidin BB, dimeric PG, benzophenone and polyphenol exhibit gastric anti-ulcer activity and cytotoxic effects towards different tumor cell lines^21^,^22^ while PG glucoside derivatives from *Conyza aegyptiaca* were found to possess antiviral and antimicrobial activities^23^,^24^.

In plant tissue culture PG showed both cytokinin-like and auxin-like activity, much like thidiazuron^25^. In addition to its role as a growth regulator, the diacetylphloroglucinol (DAPG) produced by various *Pseudomonas* biocontrol strains showed broad-spectrum activity against soil-born pathogenic bacteria, fungi and nematodes^26^,^27^,^28^,^29^. In *Eucalyptus,* formylated PG is responsible for protecting plants from insect herbivory^30^. In addition to its biological roles, PG is also used as the starting material for environmentally safe biosynthesis of the energetic material TATB that is widely used in propellants and explosive compositions^4^,^13^. Synthesis of TATB from PG would eliminate the waste streams and red water waste associated with its chemical synthesis.

Several chemical processes have been developed for the synthesis of PG and its derivatives (reviewed in ref. 9). Because of the adverse environmental impact of chemical synthesis and the goal to move towards using a sustainable resource, different bio-based approaches were explored for the synthesis of PG. The first microbial bio-based approach for PG synthesis was started after its detection in culture extract of the biological control bacterium *Pseudomonas fluorescens* Pf-5^31^. In this bacterium, biosynthesis of PG was achieved by one of the genes in a gene cluster *phlACBDE*. Three malonyl-CoA precursors are cyclized by PhlD to produce PG, which is then acylated by PhlACB to DAPG. The produced DAPG is then exported by the *PhlE*-encoded protein with the help of *PhlF*-encoded regulator^32^,^33^. Since then different genetic approaches were used to increase the amount of PG in bacteria^13^,^34^,^35^,^36^,^37^. However, to produce PG in bacteria requires an exogenous supply of glucose. Malonyl-CoA is the precursor for fatty acid synthesis and elongation^38^ as well as one of the building blocks for the biosynthesis of flavonoids and many malonylated compounds^39^. In eukaryotic cells, malonyl-CoA is formed exclusively from acetyl-CoA and bicarbonate by acetyl-CoA carboxylase (ACCase). In addition, malonyl-CoA can be made directly from malonate by malonyl-CoA synthase in Arabidopsis^40^. The presence of the precursors needed for PG synthesis in plants allows a bio-based “green synthesis” of this platform chemical by expressing bacterial *PhlD* in plants.

Here, we have generated Arabidopsis transgenic lines that express *PhlD* (either native *PhlD* from *P. fluorescens* Pf-5 (*PhlDbac*t) or a synthetic codon-optimized form (*PhlDsyn*) to enhance its expression in Arabidopsis). Both of these proteins were either retained in the cytosol (PhlD) or targeted to chloroplasts using a chloroplastic transit sequence (TP/PhlD). Our results show that transgenic lines expressing *PhlD* produce PG and over 80% of the produced PG is glycosylated to the PG-glucoside (phlorin). The highest accumulation of PG was observed in the highest expression line in which PhlD was trargeted to the chloroplast (*TP/PhlD*). A high level of PG is associated with reduced plant growth and seed viability. Also, our studies show that Arabidopsis leaves have the enzymes and the glucosyl donor necessary for glycosylation of PG.

## Results

### Generation of transgenic lines expressing the *PhlD* gene for biosynthesis of PG

**Figure 1:**
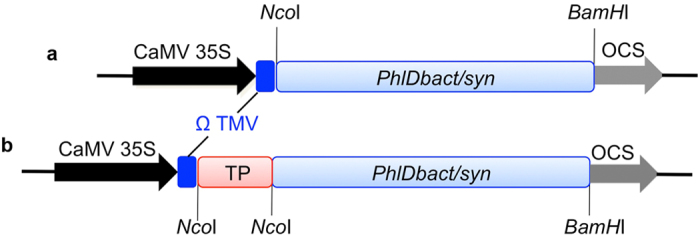
Schematic representation of gene constructs used for plant transformation. (**a**) Binary vector construct containing either bacterial (*PhlDbact*) or synthetic (*PhlDsyn) PhlD* gene under the control of Cauliflower Mosaic Virus (35S CaMV) promoter. (**b**) A similar construct like in (**a**) but the *PhlD* gene was fused to the chloroplast signal sequence (TP) of Arabidopsis NifS-like protein (*AtCpNifS*) that targets protein to chloroplasts. Restriction sites are explained in the text. OCS, octopine synthase terminator; Ω TMV, 5′ leader sequence of tobacco mosaic virus.

**Figure 2:**
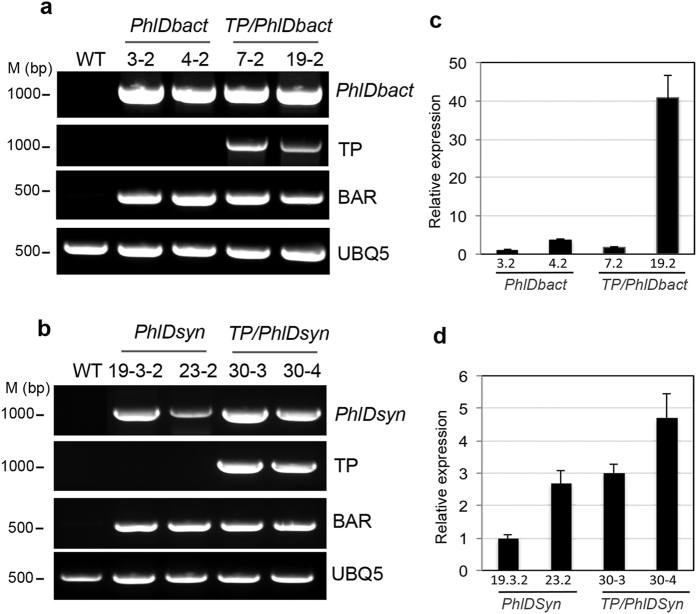
Expression analysis and quantification of *PhlD* transcript in transgenic lines. Total RNA was isolated from wild-type (WT) and transgenic lines and used for RT-PCR (left panel, a,b) and qRT-PCR (right panel, c,d). Wild-type and selection marker Basta resistance gene (BAR) were used as negative and positive controls for the transgene, respectively. Primers specific to the chloroplast transit sequence (TP) were used to confirm the presence of chloroplast targeting signal sequence. Ubiquitin (UBQ5) primers were used as an internal control for normalization. Right, (**c**) and (**d**) show quantification of PhlD transcripts in transgenic lines as relative expression. The chloroplastic 19–2 transgenic line showed the highest expression. At 40 cycles no amplification product was detected in wild-type (WT) by qRT-PCR. Gels shown are cropped. All un-cropped gels are presented as “Un-cropped Fig. 2” in “Supplementary Figures and Tables file”.

In bacteria, biosynthesis of PG from malonyl-CoA precursor requires the PhlD enzyme. We constructed a binary vector containing the *PhlD* gene from *Pseudomonas fluorescens* Pf-5 under the control of the CaMV 35S promoter (*PhlDbact*) (Fig. 1a). For efficient translation of a prokaryotic gene in eukaryotic cells, codons found in highly expressed genes in Arabidopsis were used to modify *PhlD* codons and constructed a binary vector containing a synthetic *PhlD* gene under the control of the CaMV 35S promoter (*PhlDsyn*) (Fig. 1a and Supplementary Fig. S1). Since plastid is the major site of lipid biosynthesis and malonyl-CoA is an important component of this pathway PhlD was targeted to chloroplasts to increase accumulation of PG. To target PhlD to chloroplasts the transit sequence of the chloroplastic gene *AtCpNifS* was fused to the N-terminus of PhlD (*TP/PhlDbact* and *TP/PhlDsyn*, Fig. 1b). Arabidopsis plants were transformed with either *PhlDbact; PhlDsyn; TP/PhlDbact* or *TP/PhlDsyn* and independent T1 Basta resistant plants were tested for gene insertion using genomic DNA PCR and for gene expression using RT-PCR. After two rounds of selection, two homozygous T3 lines for each construct were selected and used for further analysis. The expression of the introduced genes was confirmed by RT-PCR using sets of different primers (Fig. 2a,b) and the relative expression level among lines was measured using qRT-PCR (Fig. 2c,d). *Actin2* was used to normalize expression among different lines. No expression was observed in the wild-type whereas, the expression level of *PhlD* varied among different transgenic lines. The highest expression was observed in two lines (19–2 and 30–4) in which the *PhlD* was targeted to chloroplasts. About a 40-fold difference in expression was observed in lines with native *PhlD* whereas a 5-fold difference in expression was observed in lines where PhlD was targeted to chloroplasts (Fig. 2c,d). For brevity, plants producing PhlD in the cytosol are referred to as “cytosolic” lines whereas the lines producing PhlD in chloroplasts are referred to as “chloroplastic” lines.

### Identification of PG and phlorin in metabolite extracts of transgenic lines using mass spectrometry

**Figure 3:**
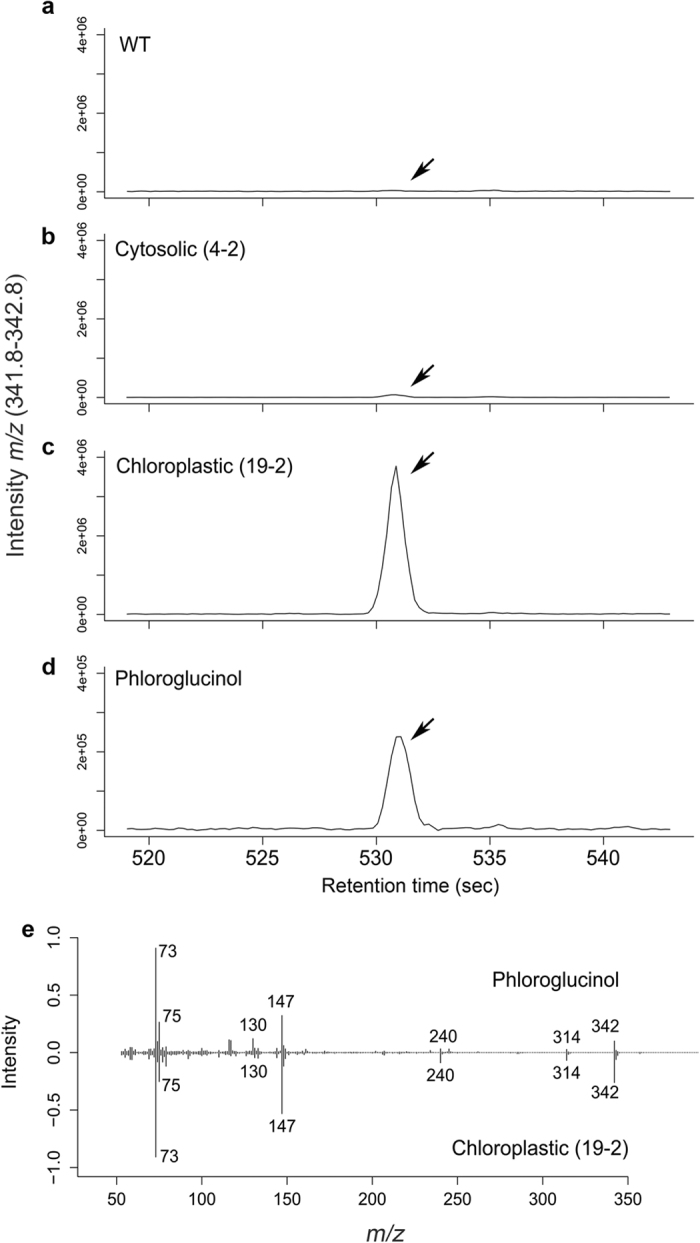
Detection of PG in transgenic lines by GC-MS. (**a–c**). Extracted ion chromatogram (EIC) of WT, cytosolic line (4–2), and chloroplastic line (19–2) extracts for *m*/*z* 342 ([M]+ for TMS3-phloroglucinol). (**d**) EIC of an authentic PG standard showing co-elution with the peak detected in transgenic lines: *m*/*z* 342 ([M]+ for TMS3-phloroglucinol). (**e**) Mass spectral match of PG standard and chloroplastic line 19–2 at 8.86 minutes demonstrating demonstrating near identical spectra between the authentic phloroglucinol and the peak produced in the chloroplastic (19–2) line.

To test the production of PG in the transgenic lines, wild-type, 4–2 (cytosolic) and 19–2 (chloroplastic) transgenic lines were grown in the soil for 3 weeks. Plant tissues were analyzed for PG using GC-MS and LC-MS. Our initial GC-MS analysis revealed a unique peak eluted at 8.86 minutes (≈530 seconds) with a characteristic *m/z* of 342, which is present predominantly in the chloroplastic (19–2) and slightly in cytosolic (4–2) transgenic lines and not in the wild-type (Fig. 3a–c). This peak co-eluted with an authentic standard of PG (Fig. 3d), suggesting that the Arabidopsis plants expressing the bacterial *PhlD* either in the cytosol or targeted to chloroplasts produced PG. Interestingly, the amount of PG detected in chloroplastic line (19–2) was higher than the cytosolic line, and this was consistent with the gene expression data (Fig. 2c). The mass spectrum of the peak detected at 8.86 minutes in the transgenic lines matched to the PG standard (Fig. 3e).

**Figure 4:**
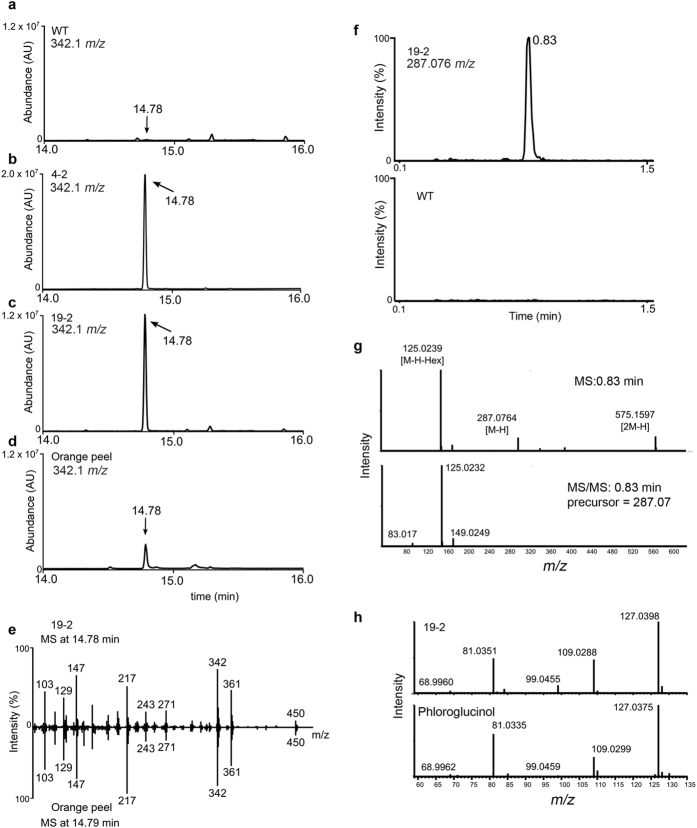
Identification of the PG conjugate in the transgenic extract by GC-MS (left) and UPLC-QTOF-MS (right). (**a–c**) A prominent *m*/*z* 342 signal was detected at 14.78 min in transgenic lines (**b**,**c**) and not in wild-type (**a**). (**d**) EIC of *m*/*z* 342 for orange peel extract supporting that orange peel has the same metabolite as that observed in the transgenic lines. (**e**) Mass spectral match for the metabolite detected at 14.78 minutes for the transgenic line 19–2 and orange peel. (**f**) UPLC-QTOF analysis of plant extracts showing a prominent signal eluted at 0.83 min with mass  *m*/*z* 287.076 in the transgenic line (upper panel) and not detected in the wild-type (bottom panel). (**g**) In-source fragmentation of the 287.076 compound showing a fragment at 125.0239, which indicates a hexose conjugate of PG. MS/MS analysis confirms that 125.0239 is a product ion of 287.0764, a pattern consistent with orange peel extract. (**h**) In source MS/MS fragmentation of putative PG conjugate from transgenic line 19–2 and PG standard in positive ionization mode showing that the in source 127.0375 fragment of PG conjugate matches that of PG standard and strongly indicate that the novel compound in transgenic plants represents a hexose conjugate of PG.

A non-targeted metabolomics analysis was conducted with these lines to identify additional metabolites that varied in the transgenic lines. A peak was detected at 14.78 minutes that was only present in transgenic lines and contained the characteristic *m*/*z* 342 (Fig. 4a–c). The elution time and mass spectrum indicated that the compound was a saccharide conjugate. A previous study^41^ has shown that plants fed with PG and radiolabelled glucose produce a PG conjugate called phlorin (phloroglucinol-β-D-glucose). To test if the saccharide conjugate is a PG glucoside, phlorin was extracted from orange peels, a tissue known to contain high levels of phlorin, and analyzed it using GC-MS. The elution time of phlorin from orange peel as well as its mass spectrum was identical to the unknown peak detected in transgenic plants (Fig. 4d,e).

An orthogonal mass spectrometry method was used to further identify this compound. The wild-type and transgenic extracts were profiled using UPLC-TOF-MS, and this compound eluted at 0.83 min (Fig. 4f). The mass spectrum of this compound suggested a molecular weight of 287.076 and generated a prominent in-source fragment of *m*/z 125.024 in negative ionization mode (Fig. 4g) and  *m*/*z* 127.02 in positive ionization mode (Fig. 4h). MS/MS analysis confirmed that the 287.0764 compound is the parent molecule of compound 125.0239 (with a confirming dimer ion at *m*/*z* = 575.1597) and indicated the presence of a hexose (287.0764 minus glucose, 162.0525). UPLC-MS/MS was performed selecting the in-source fragment ion 127.04 for MS/MS analysis, and this experiment resulted in an MS/MS spectral match between the transgenic line 19–2 and an authentic PG standard (Fig. 4h), but not an MS/MS spectrum of pyrogallol, an isomer of PG (data not shown). Taken together, these data suggest that this compound is a hexose conjugate of PG, and all evidence support the identity to be a phlorin.

**Figure 5:**
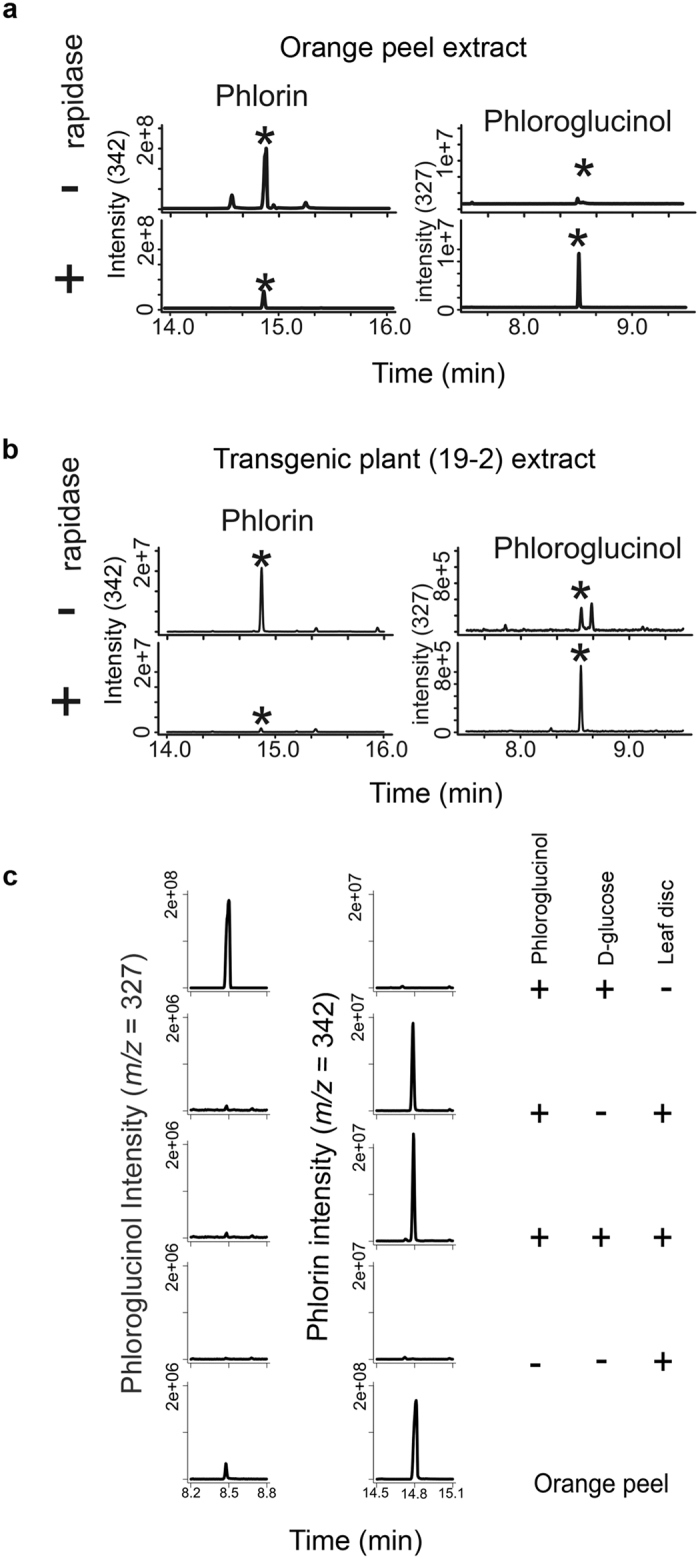
Validation of phlorin as the novel PG conjugate. (**a,b**) Phlorin extract from orange peel (**a**) and the transgenic plant (**b**) extracts were treated with β-glycosidase enzyme mix (rapidase) and resolved in GC-MS before and after digestion showing that the phlorin peak disappears and the PG peak accumulates after digestion. (**c**) Arabidopsis leaf discs were incubated with PG in the presence or absence of D-glucose, extracted and analyzed by GC-MS. Orange peel extract was used as a control to indicate phlorin formation.

To further confirm that the conjugated PG is phlorin, we digested phlorin from orange peels (Fig. 5a) and the plant extract from transgenic line 19–2 (Fig. 5b) with Rapidase (an enzyme mix that is commonly used in juice industry to increase clarity) and analyzed the products using GC-MS. Data showed that the enzyme-treated phlorin from both orange peel and plant extract is converted to PG (Fig. 5a,b). These results confirm that the conjugated compound in transgenic plants is phlorin and that a substantial amount of produced PG in transgenic lines is glycosylated to phlorin.

To test if Arabidopsis has the enzyme responsible for the *in vivo* glycosylation of PG to phlorin, wild-type Arabidopsis leaf discs were incubated with PG either in the presence or absence of D-glucose for one hour in light and analyzed the extract for the presence of phlorin using GC-MS. As a control, PG was incubated with D-glucose for the same time period and under the same conditions to test the spontaneous conversion. Phlorin was detected in all samples that had leaf discs and not detected in the control (Fig. 5c), confirming that Arabidopsis has the enzyme(s) that catalyzes the conversion of PG to phlorin. This conversion took place even in the absence of glucose, suggesting that plants have enough UDP-glucose as a substrate for glycosylation of PG.

### Quantification of phlorin in transgenic lines

**Figure 6:**
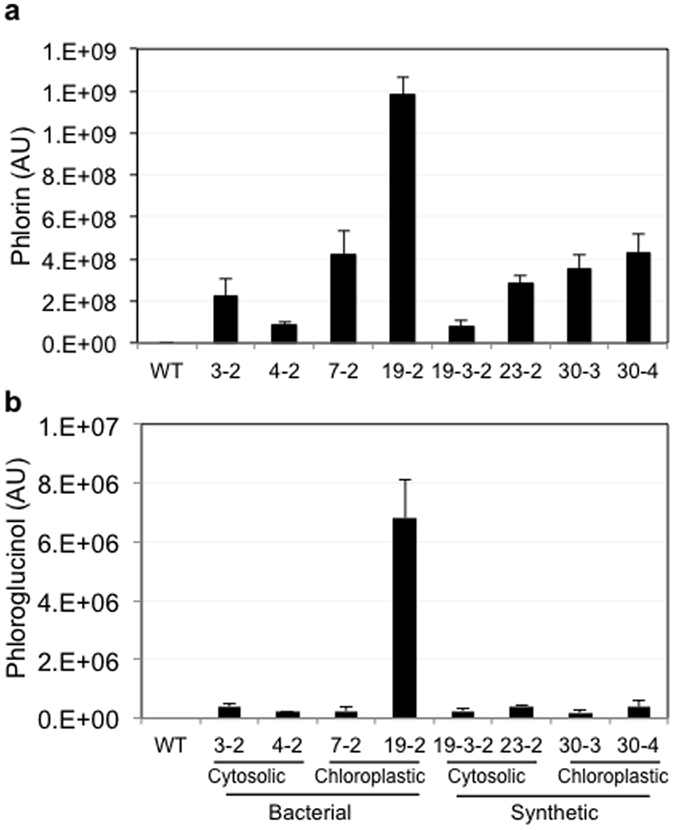
Quantification of PG and phlorin in transgenic lines. (**a**,**b**) Relative quantification of free PG (**a**) and phlorin (**b**) expressed as arbitrary units (AU).

To quantify the amount of PG and phlorin in the transgenic lines, wild-type and eight transgenic lines were grown on soil for 3 weeks. Seedlings were harvested in triplicate, ground into powder, lyophilized and used for GC-MS analysis. The relative quantification of PG and phlorin in all tested lines is presented in Fig. 6. As the phlorin standard is not available, arbitrary units (AU) were used for relative quantification of the amount of PG and phlorin among all transgenic lines. Our results showed that most of the PG is converted to phlorin (Fig. 6a,b). The amount of PG and phlorin is variable among different lines and is consistent with the gene expression data (Fig. 2), with the highest accumulation in the chloroplastic line (19–2) that showed the highest *PhlD* transcript level. Calculation of the absolute quantity of PG and phlorin, and their ratio in these lines requires authentic standards for both these chemicals. PG is commercially available, but phlorin is not. Thus, to determine the total amount of PG produced, extracts from WT and two transgenic lines (19–2 and 23–2) were treated with Rapidase and the total PG content (free and conjugate) was quantified by GC-MS using PG as a standard. Transgenic line 19–2 accumulated up to 38 mg PG/g dry tissue and about 80% of the produced PG was converted to phlorin (Table 1). In recombinant *E. coli* expressing *marA, ACCase* and *PhlD* genes the maximum amount of PG production was 0.23 g/g dry weight in a media supplemented with 20 g/L glucose^36^.

**Figure 7:**
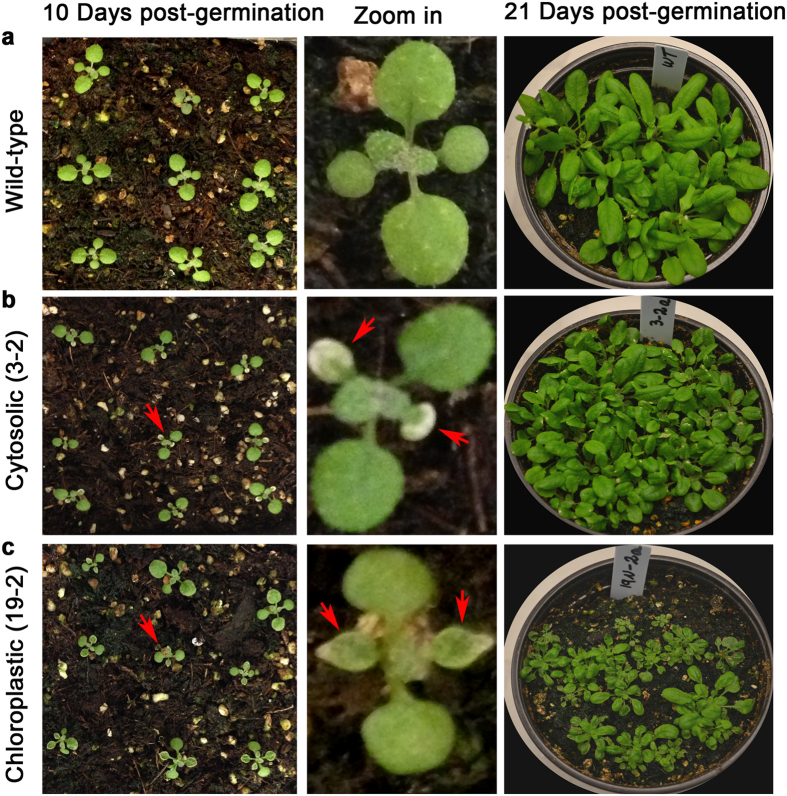
Phenotypes of chloroplastic transgenic lines. Lines 3–2 and 19–2 grown in soil showed toxicity symptoms and reduced growth as compared to wild-type (WT). Arrows indicate the zoomed in seedlings (left) and leaves showing symptoms (right).

Interestingly, the 19–2 line that showed the most PG accumulation exhibited a severe reduction in growth, general chlorosis, and thin-curled leaves as compared to wild-type (Fig. 7c). Other lines that produced less PG (e.g line 3–2) showed discoloration of only the first pair of leaves (Fig. 7b). To test if the PG affects Arabidopsis growth, wild-type Arabidopsis seedlings were grown in liquid culture at different concentrations of PG (Supplementary Fig. S2a). Seedling growth was reduced with increasing concentration of PG, suggesting that PG is toxic for plant growth. In addition to the severe growth phenotype, seeds of 19–2 are darker in color (Supplementary Fig. S2b) and showed over 50% reduction in germination (data not shown). No phenotypic differences were observed among other lines suggesting that the high accumulation of PG/phlorin in 19–2 is responsible for the growth phenotype. The deleterious effect on growth could also be related to the reduction of malonyl-CoA level in the chloroplast, which affects the lipid biosynthesis and membrane structure and/or due to the effect of accumulated metabolites.

Malonyl-CoA is mainly synthesized from acetyl-CoA by acetyl-CoA synthase (ACCase) that is present in both cytosol and plastids^42^. The plastidic pool of malonyl-CoA contributes to fatty acid synthesis, whereas the cytosolic pool is used for various biosynthetic pathways, including fatty acid elongation^38^. To test if malonyl-CoA is a limiting factor in PG biosynthesis, wild-type and transgenic lines were grown on MS medium supplemented with 2.5 mM malonate and quantified the amount of phlorin using GC-MS (Supplementary Fig. S3a). An exogenous supply of malonyl-CoA resulted in a slight reduction in phlorin, which may be due to a reduction in the growth of both transgenic and wild-type plants (Supplementary Fig. S3a,b). It was reported that exogenous malonate inhibits the growth of Arabidopsis and leads to accumulation of succinate^40^. As the malonyl-CoA pool is different in chloroplast as compared to the cytosol and the expression of ACCases is different in root and shoot^43^, we tested if the accumulation of PG is different in these tissues. Wild-type and one representative line for each construct were grown in a hydroponic system for 4 weeks (Supplementary Fig. S3c). The chloroplastic line 19–2 was not included because the growth is severely retarded in the hydroponic system. Roots and shoots were harvested separately and used for phlorin quantification using GC-MS. Higher accumulation of phlorin was obtained in roots reaching the highest amount in the chloroplastic line 7–2 (Supplementary Fig. S3d). LC-MS quantification of acetyl CoA and malonyl CoA in *Zea mays* shoot and root tissues showed that Acetyl-CoA concentrations were lower in shoots than in roots, whereas malonyl-CoA concentrations were higher in shoots than in roots^44^.

**Figure 8:**
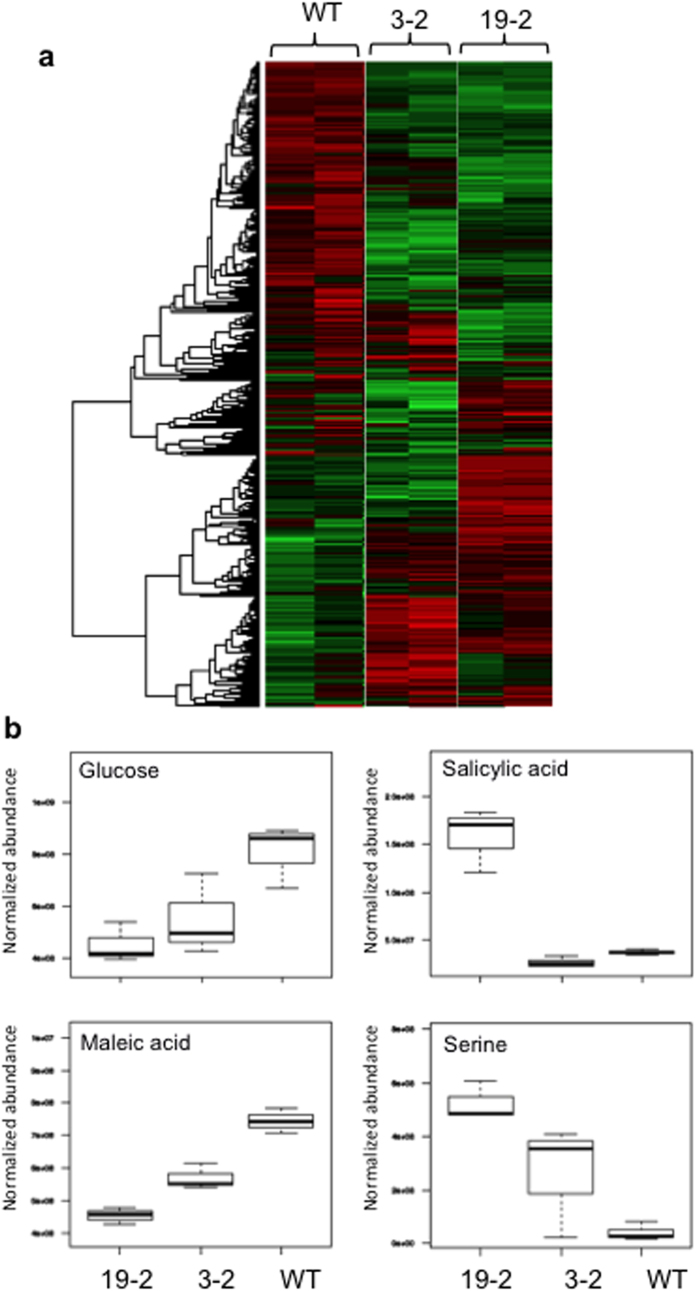
Metabolite variation in transgenic lines. (**a**) Heat map combined with hierarchical clustering of the modified GC-MS dataset for wild-type (WT), cytosolic (3–2) and chloroplastic (9–2) transgenic lines represented as a heat map. Red color indicates a higher amount of a metabolite and green color indicates a lower amount of a metabolite. (**b**) Example metabolites that are significantly altered in transgenic lines.

To identify other metabolites that varied due to *PhlD* expression, non-targeted metabolomics analysis of wild-type, one cytosolic line (3–2) and one chloroplastic line (19–2) was performed and presented the data as a heat map (Fig. 8a) (Additional File 1). This analysis has placed the wild-type, transgenic lines producing PhlD in cytosol and those lines producing PhlD in chloroplasts in three distinct groups, indicating that introduction of bacterial *PhlD* into Arabidopsis not only resulted in production of PG and phlorin, but also altered the profile of several other metabolites (Fig. 8, Supplementary Fig. S4 and Additional File 1). Among the metabolites that are significantly accumulated in the chloroplastic line and partially in cytosolic line as compared to wild-type were salicylic acid, serine, alanine, threonine, and aspartate. In contrast, the metabolites that are highly reduced in the chloroplastic lines and partially in the cytosolic line as compared to wild-type were glucose, maleic acid, linolenic acid, myo-inositol, and sinapic acid (Fig. 8b and Supplementary Fig. S4).

## Discussion

Metabolic engineering of plants using bacterial genes for production of commercially important platform chemicals, polymers and non-native proteins has been used for many years^2^,^5^,^7^,^8^,^45^,^46^,^47^. Recently, Arabidopsis plants were engineered with bacterial pathways for production of the energetic material butanetriol from either L-arabinose or D-xylose precursors^48^. Previously, rice plants were transformed with several transgenes involved in the biosynthesis of provitamin A to produce “golden rice” that has higher amounts of carotenoids in the endosperm^49^. Similar approaches have been used for the improvement of food and feed quality, increasing crop tolerance to stresses, and to develop weed and insect resistant crops (reviewed in ref. 3). Here, we report the production of PG, a platform chemical, and its glycosylated form phlorin in Arabidopsis by introducing a bacterial gene *PhlD*.

Chemical synthesis of PG is done through nitration of the petroleum-derived trinitrotoluene (TNT)^50^. Aside from the fact that TNT has explosion risks, the incomplete nitration generates toxic by-products. Other methods used to synthesize phloroglucinol from triacetic acid lactone require multiple steps^34^. Achkar *et al*.^34^, have shown that the *PhlD* gene product from *Pseudomonas fluorescens* pf-5 can convert malonyl-CoA to phloroglucinol, suggesting that bio-based methods using a single gene are possible for synthesis of phloroglucinol. Among bacteria only some species of *Pseudomonas* can synthesize PG, however, several *E. coli* strains were engineered to produce PG in culture media but this requires a continuous supply of glucose as a carbon source for generation of malonyl-CoA^34^,^36^. As plants produce malonyl-CoA, production of PG in plants would be cost effective and environmentally safe. Since codon usage is different in bacteria and plants, the expression of bacterial genes in plants might be reduced. Therefore, the codons in bacterial genes may need to be optimized for proper expression in plants^51^. In addition to native bacterial *PhlD* gene, we expressed a synthetic *PhlD* gene whose codons are optimized for expression in Arabidopsis (Fig. 1 and Supplementary Fig. S1). In plants, most of the malonyl-CoA pool is present in plastids^40^ for *de novo* synthesis of fatty acid, therefore, the chloroplast target sequence (TP) of the Arabidopsis chloroplastic gene *AtCpNiFS* was fused to the *PhlD* gene to target the enzyme to the chloroplast (Fig. 1b). In all cases, a single transcript of the expected size was observed from all constructs (Fig. 2) and accumulation of PG and phlorin were also observed (Figs 3, 4 and 5) indicating that PG can be produced in plants by expressing a bacterial gene and most of the produced PG is glycosylated to form phlorin (Table 1). The use of the synthetic *PhlD* gene did not enhance PG level as compared to the native bacterial one. Unlike most bacterial genes, the GC content of *PhlD* is high, which may have contributed to the proper expression of the native gene. As the highest PG accumulation was observed in the chloroplastic line 19–2, which showed the highest *PhlD* transcript level, it is likeky that the PG accumulation is dependent on its expression level rather than its subcellular localization.

Phloroglucinol is naturally found in certain plants such as roses, eucalyptus and acacia^52^,^53^, but nothing was detected in Arabidopsis in our analysis. In addition, more than 700 naturally occurring PG derivatives have been isolated from different systems including plants^10^ and in most cases PG is used as a building block for the synthesis of these derivatives^10^,^52^, suggesting that enzyme responsible for condensation of the precursor malonyl-CoA and cyclization into PG is present in some plants. The hydroxyl groups at alternating positions on the phloroglucinol ring are consistent with biosynthesis *via* a polyketide mechanism^33^. Three types of polyketide synthases (PKSs) are known^54^,^55^,^56^,^57^,^58^. PhlD from *Pseudomonas fluorescens* belongs to type III family of PKSs^33^. The high homology, structural similarities, and substrate specificity between PhlD and members of the CHS/STS family^33^ point to a common evolutionary origin and a similar pathway for PG synthesis in these organisms. In this regard, using enzyme preparation from parsley the PG and its derivatives were produced from a reaction of chalcone synthase substrate *p*-coumaroyl CoA and malonyl-CoA ester or other aliphatic/aromatic CoA esters^57^. Isotope tracing indicated that the PG carbon atoms are originated from three malonyl-CoA molecules through the intermediation of flavanone (naringenin)^59^ supporting the hypothesis that synthesis of PG in plants is catalyzed by type III PKSs.

Phlorin (phloroglucinol β-D-glucoside) is the simplest PG glycoside, which has been isolated from different plant species^60^,^61^. In our transgenic plants, most of the produced PG is converted to phlorin by the addition of a glycan moiety to the PG (glycosylation). The presence of glycosylation machinery in Arabidopsis was confirmed by incubating the leaf discs with PG in the presence and absence of D-glucose and detecting phlorin (Fig. 5c). In plants, glycosylation is catalyzed by glycosyl transferases such as UDP-glycosyltransferases (UGTs) that catalyze the transfer of sugar moiety from UDP-glucose to acceptor molecules such as phenylpropanoids, lipids, and proteins (reviewed in ref. 62). In the juice industry a mix of β-glycosidase enzymes (rapidase) has been used to release PG from phlorin^61^. Arabidopsis has a family of β-glycosidases that is involved in lignification^63^ but they might not have substrate specificity towards phlorin or might be located in a different cellular compartment. Introducing a phlorin-specific β-glycosidase with *PhlD* may increase the PG pool in transgenic plants.

Although *PhlD* expressing plants were able to produce PG, additional strategies are needed to further increase its level. The chloroplastic line 19–2 produced a maximum of 38 mg per gram dry tissue (Table 1). Exogenous supply of malonate did not increase PG production rather it slightly reduced it and also reduced the growth. Chen *et al*.^40^, also found that exogenous malonic acid is toxic to Arabidopsis seedlings and caused accumulation of malonic and succinic acids, a defect that can be complemented by overexpression of malonyl-CoA synthetase. Therefore, overexpression of Arabidopsis malonyl-CoA synthetase would increase the malonyl-CoA pool and also protect the plant from the toxic effect of the accumulated malonate or succinate. Another biotechnology approach that would increase the malonyl-CoA pool is by overexpression of acetyl-CoA carboxylase (ACCase). In Arabidopsis as well as other organisms the malonyl-CoA is exclusively synthesized from acetyl-CoA by acetyl-CoA carboxylase (ACCase)^40^,^42^. This should not affect the acetyl-CoA pool that is important for other metabolic processes as there are different mechanisms for generation of acetyl-CoA in different compartments^42^.

Toxicity symptoms and growth reduction associated with the metabolic engineering of plants often limit the utility of this approach in producing novel chemicals and metabolites in plants^45^,^64^. In our case, chloroplastc line 19–2 with the highest PG and phlorin showed toxicity symptoms and reduced growth (Fig. 7). Toxicity might be due to reduced level of malonyl-CoA, accumulation of intermediate metabolites and/or end products. As malonyl-CoA is the precursor for fatty acid synthesis and elongation^38^, the reduced growth might be related to changes in membrane lipid structure and content. Among the metabolites that are highly accumulated in transgenic lines, especially in 19–2, were salicylic acid and salicylic acid glucoside (Fig. 8b). Salicylic acid (free and conjugate), a key signaling molecules in plant defense against microbial attack, is known to reduce plant growth when accumulated at higher levels^65^. Arabidopsis plants lacking AtSR1 transcriptional factor accumulate high levels of free and conjugated salicylic acid and show reduced growth phenotype and early senescence^66^ like the phenotypes of the 19–2 transgenic line. It would be of interest to test the resistance of these transgenic lines to both bacterial and fungal pathogens. Further work is needed to identify the cause of toxicity. Once the cause of toxicity is identified strategies to mitigate these toxic effects could be developed. Several strategies such as engineering of these plants to increase the precursors, sequestering the product in an organelle, directing the expression to specific tissue or using transient expression systems^45^. Higher accumulation of PG was observed in root tissues (Supplementary Fig. S3), suggesting that this tissue may have a higher level of the precursor. One isoform of acetyl-CoA carboxylase that initiates at a different transcription start site and uses an alternative 5′ splice site in the first intron was accumulated to its highest level in wheat root compared to other tissues^43^, suggesting that the malonyl-CoA pool might be higher in roots of these plants. Quantification of acetyl CoA and malonyl CoA in *Zea mays* tissues showed that Acetyl-CoA concentrations were lower in shoots than in roots, whereas malonyl-CoA concentrations were higher in shoots than in roots^44^.

In conclusion, our results show that transgenic Arabidopsis plants can produce PG and its glycoside conjugate phlorin using an enzyme encoded by a bacterial gene. This study paves the way for further enhancing and optimizing production of this chemical in plants after considering metabolic bottlenecks and alleviating its toxic effect^67^. Moreover, engineering of plants for production of PG and specific derivatives such as DAPG and formylated PG should also aid in developing plants with enhanced tolerance against nematodes, herbivore and pathogenic fungi and bacteria^30^,^33^.

## Methods

### Plant materials and growth conditions

*Arabidopsis thaliana* ecotype Columbia (Col) was used in this study and all transgenic plants were generated in a Col background. For growing plants on malonate, different concentrations of malonic acid were prepared in MS medium (Cat. #2610024, MP Biomedicals)^68^ containing 1% sucrose and 0.9% phytoagar. Seeds were surface sterilized, stratified for 3 days in the dark at 4 °C, placed on agar plates and transferred to a growth chamber. All plants were grown under a 16 h light/8 h dark, 120 μmol/m^2^/s^2^ light intensity, 22 °C and 70% humidity. For growing plants in soil, seeds were stratified for 3 days, planted in soil (Metromix 250) and grown in a growth chamber under the same conditions. Tissues from aseptically grown and soil-grown plants were harvested, frozen in liquid nitrogen and stored at −80 °C for subsequent use. To test if PG is produced in roots, transgenic plants were grown in a hydroponic system. Seeds were grown vertically on MS plates for 10 days. Seedlings were moved to a hydroponic setup on 1/10X strength Hoagland’s solution^69^ and allowed to grow for 4 weeks as described^70^. Plants were harvested; roots and shoots were collected separately, frozen in liquid nitrogen and stored at −80 °C for GC-MS analysis.

### Construction of *PhlD* constructs (*PhlDbact; TP*/*PhlDbact; PhlDsyn; TP*/*PhlDsyn*)

*PhlD* gene (*locus NC_016830.1*) was PCR-amplified from *Pseudomonas fluorescens* DNA using forward and reverse primers (*PhlDbact*-F and *PhlDbact*-R, Supplementary Table S1) and PFU Ultra Hot Start Taq polymerase (Stratagene). PCR product was digested with *Nco*I/*BamH*I and ligated into the *Nco*I/*BamH*I-digested pFGC5941 binary vector (Arabidopsis Biological Resource Center, ABRC) creating *PhlDbact* construct (Fig. 1a). To target the PhlD enzyme to the chloroplast, the chloroplast targeting sequence (TP) of the chloroplast protein *AtCpNifS (At1g08490*)^71^ was PCR-amplified using sense and antisense primers (NifS-TP-F and NifS-TP-R, Supplementary Table S1), digested with *Nco*I and ligated to the 5′ end of *PhlDbact* to create *TP/PhlDbact* construct (Fig. 1b). The orientation and the nucleotide sequence were confirmed by restriction digestion and DNA sequencing, respectively.

Since codon preference could limit the expression of bacterial genes in plants *PhlD (PhlDsyn*) codons were optimized based on codon usage of the highly expressed Arabidopsis genes. Codon-optimized *PhlD* (Supplementary Fig. S1) was synthesized at GeneArt (Life Technologies, USA) and obtained as pMK-RQ/*PhlD* vector. *PhlDsyn* was amplified by PCR using forward and reverse primers (*PhlDsyn*-F and *PhlDsyn*-R, Supplementary Table S1) and pMK-RQ/*PhlD* vector as a template. Strategies used for the construction of *PhlDsyn* and *TP/PhlDsyn* were the same as native *PhlD* constructs (Fig. 1a,b).

### Generation of transgenic plants

To generate transgenic *PhlDbact, TP-PhlDbact, PhlDsyn* and *TP/PhlDsyn* plants, the pFGC5941 binary vectors harboring the genes were introduced into the GV3101 strain of *Agrobacterium tumefaciens*. Arabidopsis plants were transformed with the GV3101 strain of Agrobacterium using the floral dip method^72^. Transgenic plants were selected using BASTA (glufosinate ammonium, Sigma, USA). The homozygous T_3_ transgenic lines were used for analysis.

### RT-PCR and qRT-PCR

Total RNA was isolated from 3-week-old soil-grown seedlings using RNAeasy plant mini kit (Omega Biotech). Two μg of DNase-treated RNA was used to synthesize first-strand cDNA using a cDNA synthesis kit (Invitrogen) following the manufacturer’s instructions. For RT-PCR 1 μl of the first-strand cDNA was used in a reaction volume of 20 μl to determine the expression of the introduced genes and the fusion of the transit sequence using primers listed in the Supplementary Table S1. The expression of ubiquitin 5 (*UBQ5, At3g62250*) was used to show an equal amount of cDNA template in different samples. For qRT-PCR, primer pairs were designed using PRIMER3 software (http://frodo.wi.mit.edu) and the primer sequences are listed in Supplementary Table S1. The amplification reactions were carried out with LightCycler 480 SYBR Green 1 master mix on a LightCycler 480 (Roche Applied Science). To compare expression of the introduced genes among transgenic lines the relative expression 2^^(∆CT)^ was calculated. ∆CT was calculated by normalizing with actin transcript (*At3g18780*) (∆CT = CT _tested gene_ − CT _actin_). At amplification cycle 40 no product was detected in wild-type^73^.

### Extraction of Phlorin from orange peel

3,5-dihydroxyphenyl β-D-glucopyranoside (phlorin) was extracted from albedo layer of orange peel. One gram of albedo was ground in liquid nitrogen and then extracted with 12 ml of water for 24 h at 50 °C according to^12^. After centrifugation of the mixture, the aqueous part was concentrated under vacuum. This yielded a yellow oily residue, which was used for quantification as described.

### Synthesis of phlorin by plant tissue

Arabidopsis leaf discs (0.5 mm) were vacuum infiltrated with either 100 μM PG (Sigma-Aldrich) and 250 μM D-glucose or 100 μM PG alone or 250 μM D-glucose alone and incubated in the light for 1 h. As a control for spontaneous conversion, a mixture of PG and D-glucose is incubated in light for 1 h. Leaf discs were washed with distilled water, ground in liquid nitrogen, lyophilized and used for phlorin detection.

### Metabolite detection, phlorin and PG quantification

Frozen tissues were ground to a fine powder in a mortar and pestle and freeze-dried in a speed vacuum. One ml of methanol/water (70:30) was added to 20 mg of plant tissue and shaken on a vortex mixer for 2 h at room temperature. Samples were centrifuged at 3000 × g for 10 min at 4 °C, and 800 μl of the supernatant was transferred to a 1.5 mL microcentrifuge tube and dried in a speed vacuum. The metabolite extract was re-suspended in 50 μl of pyridine containing 25 mg/ml of methoxyamine hydrochloride, incubated at 60 °C for 45 minutes, sonicated for 10 minutes, and incubated for an additional 45 minutes at 60 °C. Next, 50 μl of *N*-methyl-*N*-trimethylsilyltri-fluoroacetamide with 1% trimethylchlorosilane (MSTFA + 1% TMCS, Thermo Scientific) was added and the samples were incubated at 60 °C for 30 minutes, centrifuged at 3000 × g for 5 min, cooled to room temperature, and 80 μl of the supernatant was transferred to a 150 μl glass insert. Metabolites were detected with a Trace GC Ultra coupled to a Thermo ISQ (Thermo Scientific), which scanned *m*/*z* 50–650  at 5 scans/sec in electron impact mode after separation on a 30 m TG-5MS column (Thermo Scientific, 0.25 mm i.d, 0.25 μm film thickness). Both the inlet and transfer line were set at 280 °C. The samples were injected in a 1:10 split ratio twice in discrete randomized blocks with a 1.2 ml/min flow rate and the program consisted of 80 °C for 30 sec, a ramp of 15 °C per min to 330 °C, and then held for 8 min. UPLC-MS/MS analysis was performed using a Waters Acquity UPLC coupled to a Waters Xevo G2 Q-TOF-MS. 1 µL of the aqueous methanol extract was injection. Separation was performed on an HSS T3 column (1.8 µM, 1.0 × 100 mm), using a gradient from solvent A (water, 0.1% formic acid) to solvent B (Acetonitrile, 0.1% formic acid). Injections were made in 100% A, held at 100% A for 1 min, ramped to 98% B over 12 minutes, held at 98% B for 3 minutes, and then returned to starting conditions over 0.05 minutes and allowed to re-equilibrate for 3.95 minutes, with a 200 µL/min constant flow rate. The column and samples were held at 50 °C and 5 °C, respectively. The column eluent was infused into a Waters Xevo G2 Q-TOF-MS with an electrospray source in positive or negative ionization mode, scanning 50-1200 m/z at 0.2 seconds per scan.

For each sample, a matrix of molecular features as defined by retention time and mass (*m*/*z*) was generated using XCMS software^74^. Samples were normalized to the total ion current and the relative quantity of each molecular feature was determined by the mean area of the chromatographic peak among replicate injections (n = 2). Mass spectra were generated using an algorithm that clusters masses into spectra (spectral clusters) based on co-variation and co-elution in the data set^75^ and searched against in-house and external databases including NIST v12, Massbank, and Metlin metabolite databases. Extracted ion chormatograms displayed in Figs 3, 4 and 5 were generated using XCMS. Phloroglucinol and phlorin chromatograms *via* GC-MS analysis shown are extracted ion chromatograms *m*/*z* as either 342.1 or 327.1+/− 0.5 Da, as depicted in each Figure, representing either 3-times TMS derivitized phloroglucinol [M]+ or M-CH3]+. UPLC-MS extracted ion chromatograms in Fig. 4f represent extracted ion chromatograms for *m*/*z* 287.076+/− 0.04 Da.

## Additional Information

**How to cite this article**: Abdel-Ghany, S. E. *et al*. Production of Phloroglucinol, a Platform Chemical, in Arabidopsis using a Bacterial Gene. *Sci. Rep.* **6**, 38483; doi: 10.1038/srep38483 (2016).

**Publisher’s note:** Springer Nature remains neutral with regard to jurisdictional claims in published maps and institutional affiliations.

## Supplementary Material

Supplementary Figures and Tables

Supplementary Information

## Figures and Tables

**Table 1:** GC-MS quantification of free PG, PG released from enzymatic digestion of phlorin and total PG (mg/g) using PG standard.

Lines	PG (mg/g lyophilized tissue)		
	Free PG	Conjugated PG (phlorin)	Total PG
19–2 (chloroplastic)	5.7 (15.2%)	31.76 (84.8%)	37.46
23–2 (cytosolic)	0.28 (23.5%)	0.91 (76.5%)	1.19

The unconjugated PG was quantified as described in the methods section. In addition, an equal amount of the extract was also digested with rapidase enzyme and the total PG was quantified. The amount of conjugated PG was calculated by subtracting the free form from the total.
