# Supplementary material for: Production of Phloroglucinol, a Platform Chemical, in Arabidopsis using a Bacterial Gene
Source: Sci Rep. 2016 Dec 7;6:38483. doi: 10.1038/srep38483 (PMC5141504; doi:10.1038/srep38483)
Supplement: Supplementary Figures and Tables [file srep38483-s1.pdf]

Supplementary Figures and Tables

Production of Phloroglucinol, a Platform Chemical, in Arabidopsis using a Bacterial Gene

Salah E. Abdel-Ghany[1,2], Irene Day[1], Adam L. Heuberger[3], Corey D. Broeckling[3] and Anireddy S.N. Reddy[1*]

[1]Department of Biology, Program in Molecular Plant Biology, Program in Cell and Molecular Biology, Colorado State University, Fort Collins, CO 80523, USA
[3]Proteomics and Metabolomics Facility, Colorado State University, Fort Collins, CO 80523, USA

Supplementary Table S1: Primers used for cloning of *PhlD* bacterial, synthetic and transit sequence, RT-PCR, and for QPCR. Restriction enzyme sequences are underlined.

Primer	Sequence (5’-3’)	Use
PhlDbact-F	CATGCCATGGCTACACTTTGCCTTCCACACGT	Cloning of PhlDbact/ PCR
PhlDbact-R	CGCGGATCCTCAGGCGGTCCACTCGCCCA	and QPCR
PhlDsyn-F	CATGCCATGGCTACTCTTTGCCTCCCTCATGT	Cloning of PhlDsyn/ RT-
PhlDsyn-R	CGCGGATCCTCAAGCAGTCCATTCTCCAACA	PCR and QPCR
NifS-TP-F	CATGCCATGGAAGGTGTGGCTATGAAACTC	Cloning of TP RT-PCR
NifS-TP-R	CATGCCATGGCGGCGGAACAAACGGATA	
Bar-F	CTGCACCATCGTCAACCA CT	Validation of T-DNA
Bar-R	TCAGATCTCGGTGACGGGCA	insertion
UBQ5-S	GGTGCTAAGAAGAGGAAGAAG	Control for RT-PCR
UBQ5-AS	CTCCTTCTTTCTGGTAAACG	
Actin-F	GGCAAGTCATCACGATTGG	Control for qRT-PCR
Actin-R	CAGCTTCCATTCCCACAAAC	

Supplementary Figure S1. Alignment of *PhlDbact* (Bacterial) and *PhlDsyn* (Synthetic).
Chloroplast targeting sequences are underlined. Start and stop codons are highlighted.
Nucleotides that are changes in the synthetic *PhlD* are highlighted in red.

```
Bacterial    ATGGCGGCGGAACAAACGGATAGCGAAGAGGAGCAACGAACGCGGGAGAAGCTCCGGTGG

Synthetic    ATGGCGGCGGAACAAACGGATAGCGAAGAGGAGCAACGAACGCGGGAGAAGCTCCGGTGG


Bacterial    CCGATGGAGATGGCGTTAGGGAACGACGGGAGTTTCATAGCCACACCTTCCATGTCTACA

Synthetic    CCGATGGAGATGGCGTTAGGGAACGACGGGAGTTTCATAGCCACACCTTCCATGTCTACT


Bacterial    CTTTGCCTTCCACACGTCATGTTTCCGCAACACAAGATCACCCAGCAACAGATGGTCGAT

Synthetic    CTTTGCCTCCCTCATGTTATGTTCCCTCAGCACAAGATCACTCAACAGCAGATGGTTGAT


Bacterial    CACCTGGAAAACCTGCACGCCGACCATCCACGCATGGCCCTGGCCAAGCGCATGATCGCC

Synthetic    CATCTTGAGAACCTCCATGCTGATCATCCTAGAATGGCTCTCGCTAAGAGAATGATCGCT


Bacterial    AACACCGAAGTCAACGAGCGCCACCTGGTGTTGCCGATCGACGAACTGGCAGTGCACACC

Synthetic    AACACTGAGGTTAACGAGAGACATCTTGTGCTTCCAATCGATGAGCTTGCTGTGCATACT


Bacterial    GGTTTCACCCACCGCAGCATCGTCTACGAGCGTGAAGCCCGGCAGATGTCGTCGGCCGCG

Synthetic    GGATTCACCCATAGATCTATCGTTTACGAGAGAGAGGCTAGACAAATGTCATCTGCTGCT


Bacterial    GCGCGCCAGGCCATCGAGAATGCCGGGCTGCAGATCAGCGACATTCGCATGGTGATCGTC

Synthetic    GCTAGACAAGCTATTGAGAACGCTGGACTCCAAATCTCTGATATCAGGATGGTGATCGTG


Bacterial    ACTTCCTGCACCGGCTTCATGATGCCGTCGCTGACCGCGCACCTGATCAACGACCTGGCC

Synthetic    ACTTCTTGTACCGGATTCATGATGCCTTCTCTCACTGCTCATCTCATCAACGATCTTGCT


Bacterial    CTGCCAACCTCCACCGTGCAGTTGCCGATCGCCCAGCTGGGCTGCGTGGCCGGTGCCGCG

Synthetic    CTCCCTACTTCTACTGTTCAACTCCCTATCGCTCAACTTGGATGTGTTGCTGGTGCTGCT


Bacterial    GCCATCAACCGCGCCAACGACTTCGCCCGGCTCGATGCCCGCAACCACGTACTGATCGTG

Synthetic    GCTATTAACAGGGCTAACGATTTCGCTAGACTCGATGCTAGAAACCATGTTCTCATCGTG


Bacterial    TCCCTGGAATTCTCCTCGCTGTGCTACCAGCCGGACGACACCAAGCTGCACGCCTTCATC

Synthetic    TCTCTCGAGTTCTCTTCTCTTTGCTACCAACCTGATGATACAAAGCTCCACGCTTTCATT
```

**Bacterial**	**TCCGCGGCGCTGTTCGGCGATGCGGTATCCGCCTGCGTGCTGCGCGCCGATGACCAGGCC**
**Synthetic**	**TCTGCTGCTTTGTTCGGAGATGCTGTTTCTGCTTGTGTTCTCAGAGCTGATGATCAAGCT**
**Bacterial**	**GGCGGCTTCAAGATCAAGAAGACCGAGTCGTACTTCCTGCCCAAGAGCGAGCACTACATC**
**Synthetic**	**GGTGGATTCAAGATCAAGAAAACCGAGTCTTACTTCTTGCCTAAGTCTGAGCACTACATC**
**Bacterial**	**AAGTACGACGTGAAGGACACCGGCTTTCACTTCACCCTCGACAAGGCGGTGATGAACTCC**
**Synthetic**	**AAGTACGATGTGAAGGATACTGGATTCCACTTCACTCTCGATAAGGCTGTGATGAACTCT**
**Bacterial**	**ATCAAGGACGTGGCACCGGTCATGGAGCGGCTCAACTACGAGAGCTTCGAACAGAACTGT**
**Synthetic**	**ATCAAGGATGTTGCTCCTGTTATGGAAAGACTCAACTACGAGTCTTTCGAGCAAAACTGC**
**Bacterial**	**GCGCACAACGACTTCTTCATCTTCCACACCGGTGGTCGCAAGATCCTCGACGAGCTGGTG**
**Synthetic**	**GCTCATAACGATTTCTTCATCTTCCACACCGGTGGAAGAAAGATTCTTGATGAGCTTGTG**
**Bacterial**	**ATGCACCTGGACCTGGCATCCAACCGGGTCTCGCAATCGCGCAGCAGCCTGTCGGAAGCC**
**Synthetic**	**ATGCATCTTGATCTCGCTTCTAACAGAGTGTCTCAGTCTAGATCTTCTTTGTCTGAGGCT**
**Bacterial**	**GGCAACATTGCCAGCGTGGTGGTGTTCGACGTACTCAAGCGGCAGTTCGATTCCAACCTC**
**Synthetic**	**GGAAACATTGCTTCTGTGGTGGTTTTCGATGTTCTCAAGAGGCAGTTCGATTCTAACCTC**
**Bacterial**	**AATCGCGGCGACATCGGCCTGCTGGCAGCCTTCGGCCCGGGGTTCACCGCGGAAATGGCG**
**Synthetic**	**AACAGAGGTGATATTGGACTTCTTGCTGCTTTTGGACCTGGATTCACTGCTGAAATGGCT**
**Bacterial**	**GTGGGCGAGTGGACCGCCTGA**
**Synthetic**	**GTTGGAGAATGGACTGCTTGA**

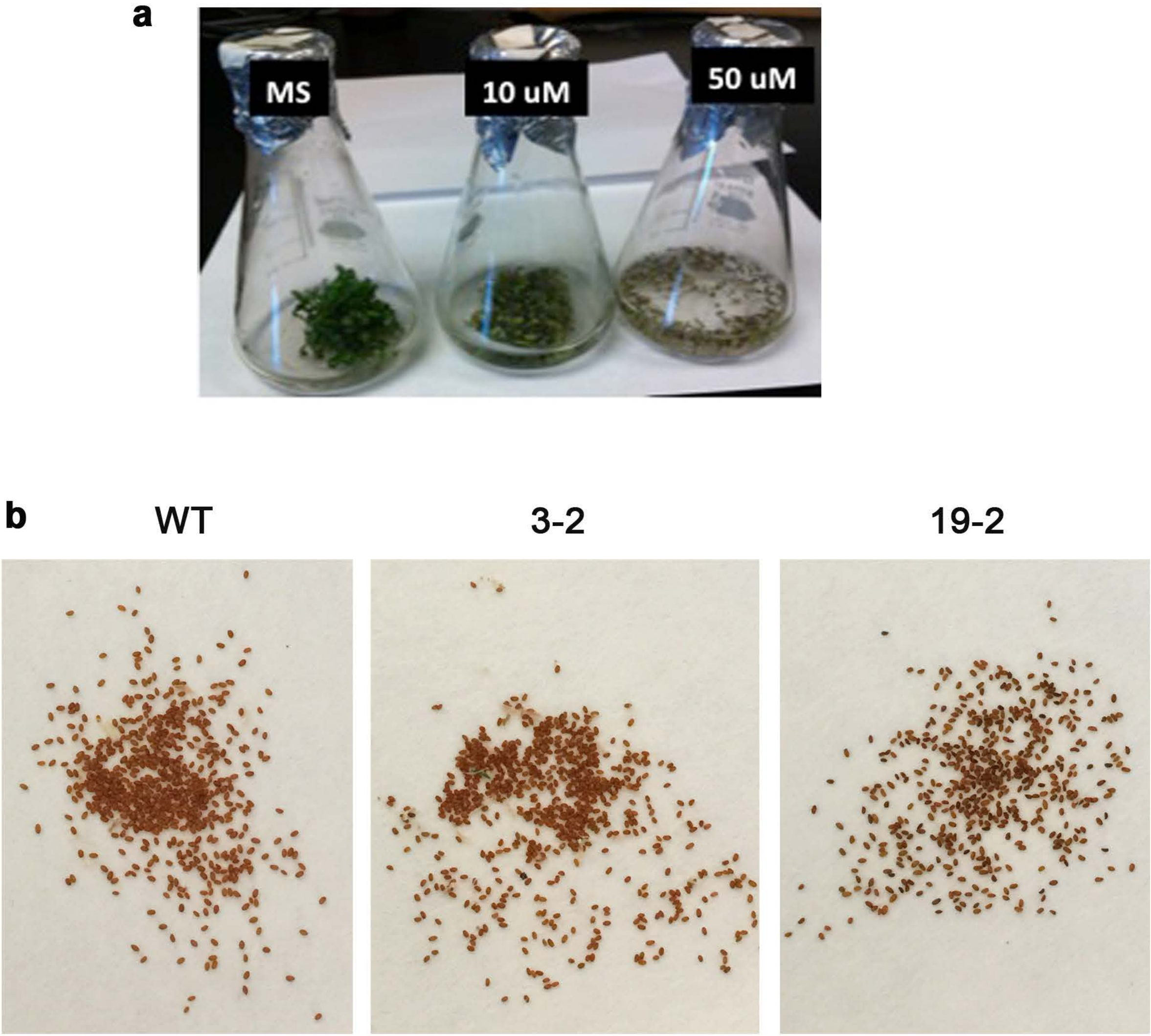

Supplementary Figure S2. Effect of PG on Arabidopsis growth (**a**) and seed color (**b**) of PhlD-expressing plants. WT, Wild-type; 3-2, Transgenic line expressing PhlD in cytosol; 19-2, Transgenic line in which PhlD is targeted to chloroplasts.

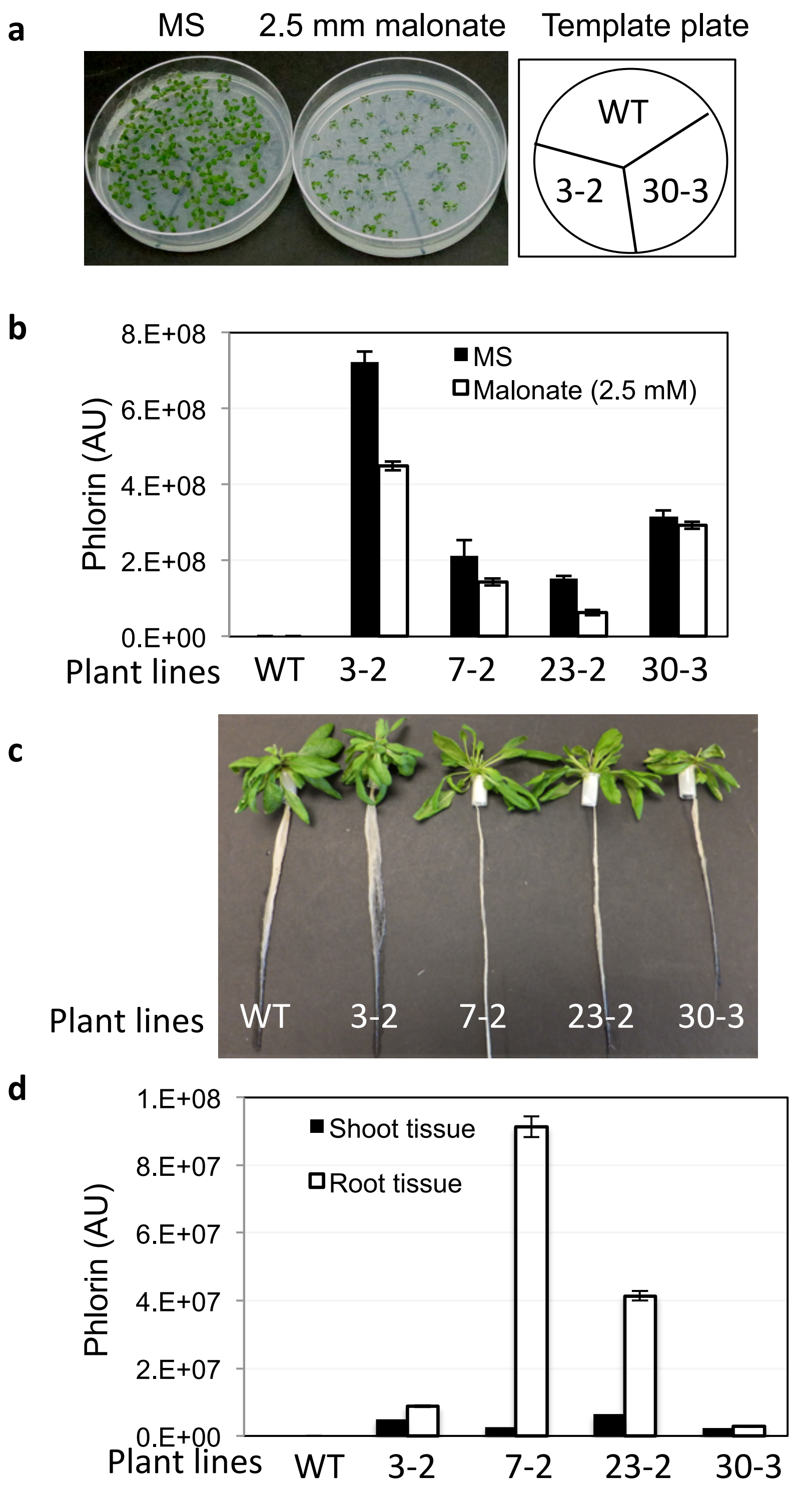

Supplementary Figure S3. Growth and quantification of phlorin in presence and absence of malonate (a,b) and in roots and shoots harvested from hydroponic growing seedlings (c,d). Wild-type and transgenic lines were grown on either MS and or MS supplemented with 2.5 mM malonate (**a**) and seedlings were used to quantify phlorin (**b**). For quantification of phlorin in roots and shoots seedlings of wild-type and transgenic lines were grown in hydroponic system (**c**) and tissues were harvested separately and used for phlorin estimation (**d**). Phlorin vales were expressed as arbitrary units (AU). Data are average of three replicates and error bars represent the standard deviation.

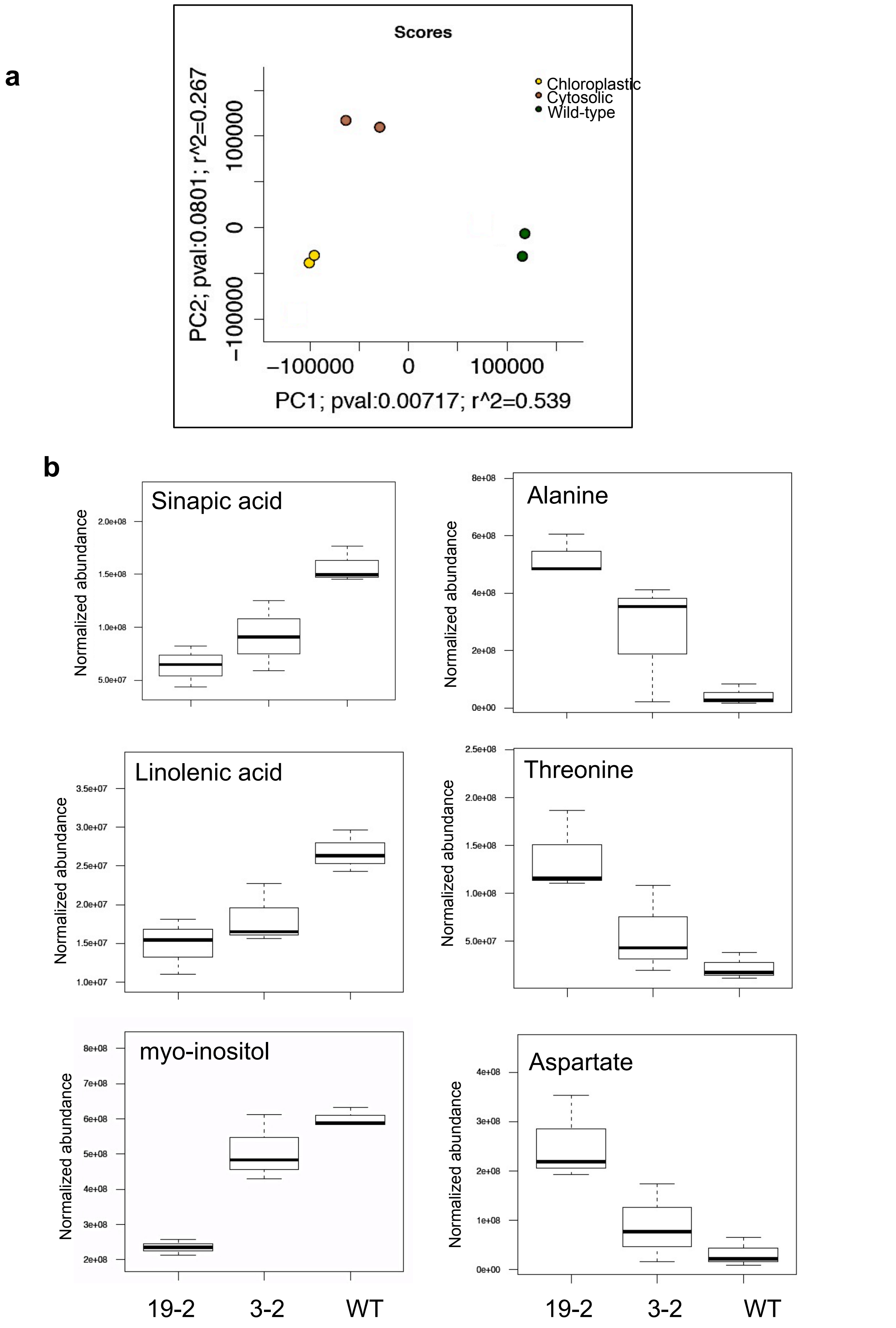

Supplementary Figure S4. Metabolic profiling of transgenic lines. (**a**) Principal component analysis of the modified GC-MS dataset for wild-type (WT), cytosolic (3-2) and chloroplastic (9-2) transgenic lines. (**b**) Examples of some metabolites that are significantly altered in transgenic lines.

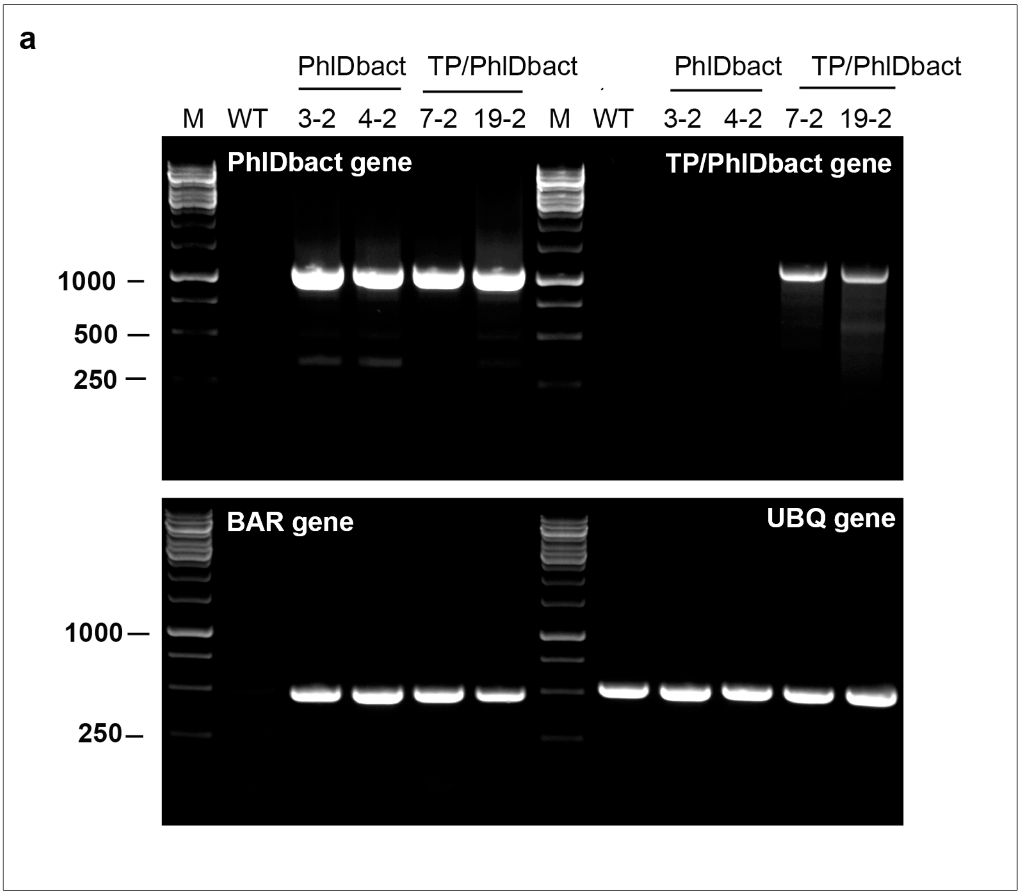

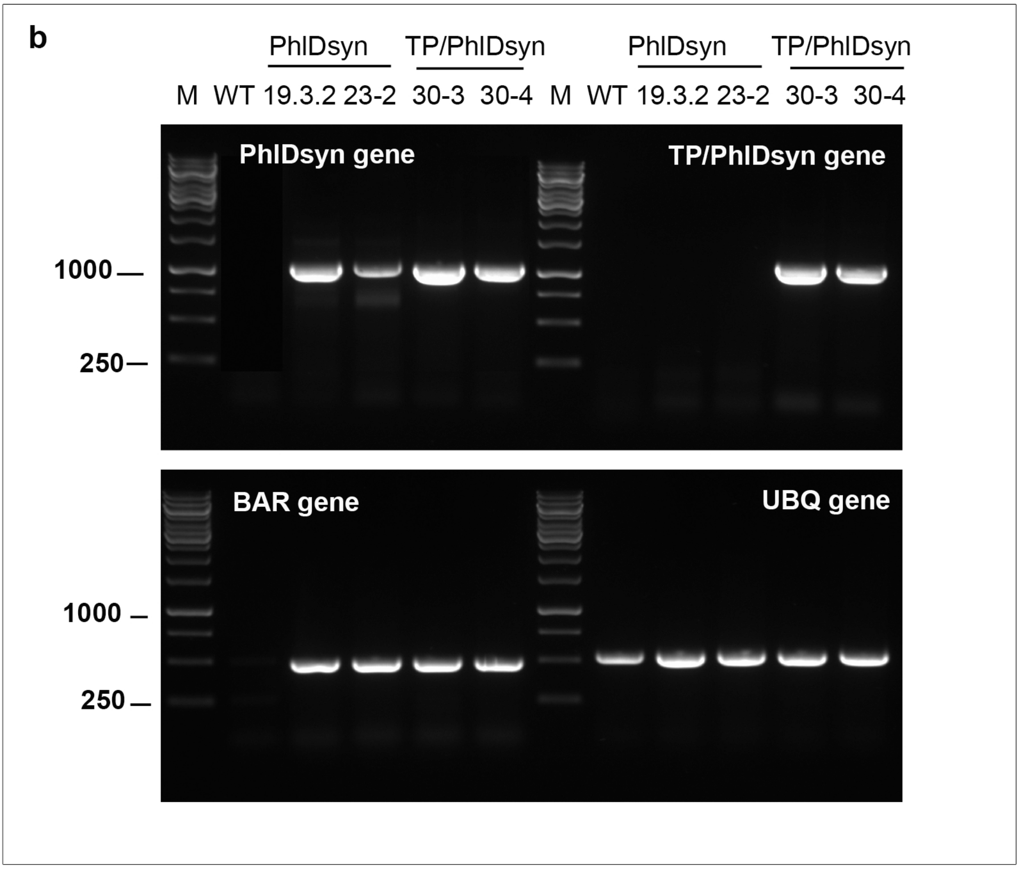

Uncropped Figure 2

Uncropped images used to prepare Figure 2
